# Cancerouspdomains: comprehensive analysis of cancer type-specific recurrent somatic mutations in proteins and domains

**DOI:** 10.1186/s12859-017-1779-5

**Published:** 2017-08-16

**Authors:** Seirana Hashemi, Abbas Nowzari Dalini, Adrin Jalali, Ali Mohammad Banaei-Moghaddam, Zahra Razaghi-Moghadam

**Affiliations:** 1 College of Science, University of Tehran, Tehran, Iran; 2 Max Planck Institute for Informatics, Saarland Informatics, Campus, 66123 Saarbrücken, Germany; 3 Institute of Biochemistry and Biophysics (IBB), University of Tehran, Tehran, Iran; 4 Faculty of New Sciences and Technologies, University of Tehran, North Kargar St, Tehran, Tehran 1439957131 Iran

**Keywords:** Cancer, Protein domain, Pfam, Cath, Pan-cancer, Somatic mutation, TCGA exome sequencing data

## Abstract

**Background:**

Discriminating driver mutations from the ones that play no role in cancer is a severe bottleneck in elucidating molecular mechanisms underlying cancer development. Since protein domains are representatives of functional regions within proteins, mutations on them may disturb the protein functionality. Therefore, studying mutations at domain level may point researchers to more accurate assessment of the functional impact of the mutations.

**Results:**

This article presents a comprehensive study to map mutations from 29 cancer types to both sequence- and structure-based domains. Statistical analysis was performed to identify candidate domains in which mutations occur with high statistical significance. For each cancer type, the corresponding type-specific domains were distinguished among all candidate domains. Subsequently, cancer type-specific domains facilitated the identification of specific proteins for each cancer type. Besides, performing interactome analysis on specific proteins of each cancer type showed high levels of interconnectivity among them, which implies their functional relationship. To evaluate the role of mitochondrial genes, stem cell-specific genes and DNA repair genes in cancer development, their mutation frequency was determined via further analysis.

**Conclusions:**

This study has provided researchers with a publicly available data repository for studying both CATH and Pfam domain regions on protein-coding genes. Moreover, the associations between different groups of genes/domains and various cancer types have been clarified. The work is available at http://www.cancerouspdomains.ir.

**Electronic supplementary material:**

The online version of this article (doi:10.1186/s12859-017-1779-5) contains supplementary material, which is available to authorized users.

## Background

Cancer refers to a group of diseases characterized by uncontrolled growth and division of cells in the body, and is caused by environmental as well as genetic factors. Genetic factors include, but are not limited to inherited germline mutations, changing DNA methylation rate and microRNA modifications. Cancer is a leading cause of death in most countries. The number of new cases of cancer is 454.8 per 100,000 incidents per year and the number of cancer deaths is 171.2 per 100,000 incidents per year [1–4]. Accordingly, developing methods for detection and treatment of cancer is a main area of interest as well as a challenge.

Several studies have been conducted to find genes that are involved in cancer development [5–8]. Even though there has been some degree of success in identifying genes that are strongly associated with cancer, much is yet to be done for discovering causal genes and variants. In addition, most of those studies disregard the position of those mutations, whereas mutations at different positions of a certain gene may lead to various levels of damage [9, 10].

Proteins are responsible for most cellular functions and their malfunction may undermine cellular performance [11]. Only some of the mutations in coding regions, and not all of them lead to cancer. Therefore, distinguishing mutations with drastic impacts on protein functionality may help discriminate driver mutations from less significant ones. To this end, some researches have focused on mapping genomic positions to protein sequences and tried to distinguish mutations that affect the functionality of proteins [10, 12]. Protein domains are conserved regions of proteins that can fold and act independently [13]. Therefore, it is plausible that mutations within these regions may cause more damage compared to other mutations [13]. To this aim, some efforts have been made to study cancer mutations at the protein domain level. Nehrt et al. [12] mapped non-synonymous somatic mutations of *Breast Invasive Carcinoma* and *Colon Adenocarcinoma Tumor* samples to their corresponding protein domains, in order to extract domains with significant mutation frequency. In another study by Yang et al. [10], mutational protein domain hotspots for 21 different cancer types were determined by mapping somatic mutations to protein domains. Regions with high numbers of mutation for each cancer type were called hotspot.

This study represents a method to explore protein domains with significant mutation frequencies, using whole exome sequencing data. Beside analyzing Pfam protein domains as sequence-based domains, CATH protein domains have also been studied as structure-based domains, which were not included in relevant studies to this date. Moreover, in order to more specifically pinpoint the domains of each cancer type, 29 different cancer types as well as pan-cancer were investigated in this study. In addition, the frequency of mutations in mitochondrial genes, stem cell-specific genes and DNA repair genes were examined. These sets of genes are likely to have important roles in cancer development and progression. Furthermore, the interconnectivity of proteins with mutation on causal domains was investigated.

## Methods

### Data extraction

Whole-exome sequencing data of 7685 cancer patients from 29 different cancer types containing 2,057,977 somatic mutations are downloaded from the TCGA (The Cancer Genome Atlas) data portal [14]. The detailed list of cancer types as well as the number of patients for each type is shown in Table 1. The names of downloaded files (in July 2015) for each cancer type is shown in Additional file 1: Table A1. The data are extracted from non-metastatic patients before giving radiotherapy or chemotherapy and are mapped to the human genome references of the GRCh37 [15].

**Table 1:** Prevalence of patients, mutations and domain-specific mutations in different cancer types

Cancer type	Abbrevation	patients	Somatic Mutations	Mutations on Protein Coding Regions	Mutations on Pfam Domains	Mutations on CATH Domains
Adrenocortical carcinoma	ACC	451	22,679	21,355	8335	1090
Bladder Urothelial Carcinoma	BLCA	169	169,165	154,893	69,727	11,081
Breast invasive carcinoma	BRCA	983	98,882	93,405	40,390	6512
Cholangiocarcinoma	CHOL	460	8593	7030	3025	484
Colon adenocarcinoma	COAD	412	125,386	123,917	55,913	8055
Esophageal carcinoma	ESCA	202	79,536	67,566	29,249	4340
Glioblastoma multiforme	GBM	92	20,932	20,225	9905	1692
Head and Neck squamous cell carcinoma	HNSC	545	151,456	131,314	59,768	9301
Kidney Chromophobe	KICH	178	8739	8023	3425	489
Kidney renal clear cell carcinoma	KIRC	36	51,235	47,964	21,059	3404
Kidney renal papillary cell carcinoma	KIRP	269	33,247	31,433	13,530	2074
Brain Lower Grade Glioma	LGG	182	47,286	42,890	19,750	3489
Liver hepatocellular carcinoma	LIHC	230	89,042	82,443	36,749	5470
Lung adenocarcinoma	LUAD	275	242,542	216,730	101,616	13,522
Lung squamous cell carcinoma	LUSC	171	65,306	63,981	30,542	4206
Ovarian serous cystadenocarcinoma	OV	524	12,751	12,214	5824	998
Pancreatic adenocarcinoma	PAAD	175	82,871	72,462	29,279	4223
Pheochromocytoma and Paraganglioma	PCPG	425	8706	7629	3115	472
Prostate adenocarcinoma	PRAD	116	26,862	24,094	10,954	1657
Rectum adenocarcinoma	READ	254	34,260	33,885	15,774	2313
Sarcoma	SARC	75	83,019	71,650	30,021	4526
Skin Cutaneous Melanoma	SKCM	387	53,086	49,645	23,347	2993
Stomach adenocarcinoma	STAD	144	223,884	213,642	96,340	14,143
Testicular Germ Cell Tumors	TGCT	356	30,293	26,170	10,902	1518
Thyroid carcinoma	THCA	123	16,807	15,383	7245	1243
Thymoma	THYM	248	39,467	35,892	15,655	2379
Uterine Corpus Endometrial Carcinoma	UCEC	66	212,745	204,826	93,937	13,959
Uterine Carcinosarcoma	UCS	57	14,212	11,803	5266	876
Uveal Melanoma	UVM	80	4988	4411	2087	313
pan-cancer		7685	2,057,977	2E + 06	852,729	126,822

Since we are interested in discovering the role of protein domains in cancer, only protein-coding genes were selected among genes reported in TCGA data. Among 2,057,977 somatic mutations reported in this database, 1,896,875 of them occurred in protein-coding regions. Given that synonymous mutations have no effect on protein sequence and no demonstrable impact on phenotype [16, 17], in this study, only non-synonymous mutations within protein coding regions are considered.

Protein domains can be defined in two different ways, either by their sequences or by their structural characteristics. Both these definitions are considered in this study in order to better understand the role of domains in cancer. Pfam [18, 19] and CATH [20, 21] databases are used to extract sequence-based and structure-based domains, respectively, and the UCSC (University of California Santa Cruz) [22] tables and PDB [23] database are exploited to extract the start and end positions of coding regions, exons, and more specifically, domains in genome.

HUGO (HUman Genome Organization) [24] standard gene nomenclature is employed to identify protein-coding genes. The number of protein-coding genes in HUGO is 19,011, all except for 22 of which are linked to PDB and Pfam entries, and 10,913 of them have Pfam domains. All predicted Pfam domains, without any constraints on *E*-value and bit-rate, were extracted in this study. A CATH domains was selected if it is represented by the same exact sequence in a UniProt record. To map CATH domains position form PDB residue to UniProt sequence, we used SIFTS [25], which is a manually curated database to match the positions of PDB entries to UniProt sequences.

### Identification of candidate domains and genes

Once data have been acquired and evaluated, the next step was to extract candidate regions by use of statistical analysis. Candidate regions (domains or genes) are regions in which mutations occur more frequently than expected. If mutations are mutually independent and uniformly distributed over the combined sequences of coding regions within human genome, then for each mutation, the probability of occurring on the *i* ^th^ coding region is *p*_*i*_, which is equal to the length of *i* ^th^ coding region, *l*_*i*_, divided by total length of coding regions, *L*, in the whole genome, that is, ${p}_i=\frac{l_i}{L}$. To extract candidate regions at domain level, *l*_*i*_ and *L* are respectively set to be the length of *i* ^th^ domain and the total length of domains in the whole proteome.

Suppose that *n* is the number of mutations occurred in all protein-coding regions and *k*_*i*_ is the number of mutations happened in the *i* ^th^ coding region, then the probability of having *k*_*i*_ mutations on the *i* ^*th*^ coding region can be modeled by the binomial distribution, as follows [26].

1 $$ \mathit{\Pr}\left(X={k}_i\right)=\left(\genfrac{}{}{0pt}{}{n}{k_i}\right){p}_i^{k_i}{\left(1-{p}_i\right)}^{\left(n-{k}_i\right)}=\left(\genfrac{}{}{0pt}{}{n}{k_i}\right){\left(\frac{l_i}{L}\right)}^{k_i}{\left(1-\frac{l_i}{L}\right)}^{\left(n-{k}_i\right)} $$

To determine whether a protein-coding region is a potential candidate for a specific cancer, the number of observed mutations on that region is compared with what would be expected by the binomial distribution model, and a *p*-value threshold of 0.05 is adopted to test the null hypothesis [27]. To this aim, for *k* observed mutations on each region, the hypothesis is rejected if *p*(*X* < *k*) > 0.95, where

2 $$ P\left(X<k\right)=\sum \limits_{j=0}^{k-1}\mathit{\Pr}\left(X=j\right)=\sum \limits_{j=0}^{k-1}\left(\genfrac{}{}{0pt}{}{n}{j}\right){p}^j{\left(1-p\right)}^{n-j} $$

Since multiple independent hypothesis tests are conducted in all cases, to maintain the family-wise error rate (FWER) [27], a post hoc Bonferroni [28] test is applied. Accordingly, when *m* independent hypothesis tests are performed, the criterion for rejecting the null hypothesis is divided by *m*. In other words, when the significance level for the whole family of tests is set to be 0.05, then with Bonferroni correction each individual hypothesis is evaluated at a significance level of$\frac{0.05}{m}$.

To eliminate the possibility of overflow or underflow in computing values such as$\left(\genfrac{}{}{0pt}{}{n}{k}\right)$, ${\left(\frac{l}{L}\right)}^k$ and ${\left(1-\frac{l}{L}\right)}^{\left(n-k\right)}$, log Pr (*X* = *k*) is calculated instead of Pr(*X* = *k*). Accordingly, computations are performed using eq. 3 instead of eq. 1:

3 $$ \mathit{\log}\mathit{\Pr}\left(X=k\right)=\mathit{\log}\left(\genfrac{}{}{0pt}{}{n}{k}\right)+k\mathit{\log}p+\left(n-k\right)\mathit{\log}\left(1-p\right) $$

In addition, to avoid numerical problems in computing $\left(\genfrac{}{}{0pt}{}{n}{k}\right)$ in eq. 3, Stirling’s approximation [29] is applied.

### Aims and objectives of the study

There are more than 100 types of cancer [30] and despite their differences, they present underlying biological (genetic) similarities. Pan-cancer study aims to uncover similarities and differences between various cancer types [31]. With this background, all the data downloaded from different cancer types are assembled together in this study to form a pan-cancer dataset for further investigations.

The main focus of this study is to assess the frequency of mutations on domain regions. However, we are also interested in evaluating the frequency of mutations in protein coding regions of three particular sets of genes: mitochondrial genes, stem cell-specific genes and DNA repair genes. Mitochondria are responsible for producing energy in almost all cell types and have their own DNA. Since mitochondrial DNA mutations are known to be highly associated with human cancer [32], mutations within the mitochondrial genome are investigated in this study. Most of cancerous cells possess the classical characteristics of normal stem cells, including extensive capacity of self-renewal and acquired resistance to apoptosis [33, 34]. Therefore, genes responsible for the maintenance of stem cells are appropriate candidates for our goal. Mutation in genes that are associated with DNA repair function in a cell may induce partial loss of gene functionality [35, 36]. In this light, studying the presence of mutations in these genes may also be informative for cancer research.

## Results and discussion

This study covers four areas of assessment, namely, mutations in protein coding regions of mitochondrial, stem cell-specific and DNA repair genes, and mutations in protein domain regions. The results of each assessment are described in the following subsections.

### Mitochondrial genes

Several studies have reported the presence of somatic mitochondrial mutations in cancer cells. Even though many of these studies have demonstrated the role of mitochondrial mutations in human cancers such as *Kidney Renal Cell Carcinoma* [37], *Breast Invasive Carcinoma* [38], *Gastric Carcinoma* [39], *Prostate Adenocarcinoma* [40], *Ovarian Carcinoma* [41] and *Thyroid Carcinoma* [42], such an association was not identified in all relevant studies. For instance, studies on *Glioblastoma Multiforme* [43] and *Liver Hepatocellular Carcinoma* [44] have not been able to pinpoint the role of mitochondrial mutations in cancer. In this light, it is worthwhile to further investigate the role of mitochondria in cancer development.

To examine the role of mitochondria in cancer development, the observed somatic mutations in all mitochondrial genes are studied. There are 37 different genes in mitochondrial DNA, which are assigned to six groups of complexes, based on their roles (shown in Additional file 1: Table A2). For instance, the genes MT-RNR1 and MT-RNR2, which are responsible for making rRNAs, are assigned to rRNA complex [45]. Mutations within each group are identified to better understand its role in developing cancer.

To identify mitochondrial candidate genes associated with each of the 29 cancer types as well as pan-cancer (30 cancer types in total), the statistical analysis is performed on two levels. In the first level of analysis, each mitochondrial gene is considered individually, while in the second level, genes are analyzed in their group (complex) context. Accordingly, in the Bonferroni correction, the parameter *m* is set to 37 × 30 and 6 × 30 for the first and second level, respectively. With a corrected *p*-value threshold of $\frac{0.05}{m}$, there are 13 cancer types and pan-cancer (shown in Table 2) for which at least one mitochondrial candidate gene or complex is identified. In Table 2, the number in parentheses next to each gene shows the percentage of patients for which this gene is mutated. All in all, nine mitochondrial genes have been identified as candidate ones: MT-CO2, MT-CYB, MT-ND1, MT-ND5, MT-RNR1, MT-RNR2, MT-TL1, MT-TT and MT-TV. Additional file 1: Table A3 shows the number of patients with mitochondrial mutations and the number of mutations for each.

**Table 2:** Candidate mitochondrial genes and complexes for each cancer type

Cancer Type	Genes (Percentage)	Complexes (Percentage)
ACC	MT-TL1 (32.6)	-
BRCA	MT-RNR1 (3.6), MT-RNR2 (5.6), MT-TT (0.8)	rRNA (8.4)
CHOL	MT-RNR2 (33.3)	rRNA (33.3)
ESCA	MT-CYB (13.7)	COMPLEX III (13.7)
HNSC	MT-ND5 (1)	-
KICH	MT-ND1 (13.6)	-
LIHC	MT-ND5 (12.9)	COMPLEX I (18.3)
LUAD	-	COMPLEX III (1.1)
PRAD	MT-RNR2 (1.9)	-
SARC	MT-RNR2 (12.6), MT-CO2 (7.1)	rRNA (14.2), COMPLEX IV (15.7)
TGCT	-	COMPLEX III (6.3)
THYM	MT-RNR2 (27.6)	rRNA (27.6)
UCEC	MT-RNR1 (11.7), MT-RNR2 (11.7), MT-TV (12)	rRNA (0.41.7)
Pan Cancer	MT-RNR2, MT-CYB	rRNA, COMPLEX III

Among six mitochondrial groups of complexes, ATP synthesis and tRNA complexes have not been chosen as candidate for any cancer type. In particular, no significant mutation was observed in genes MT-ATP6 and MT-ATP8 in any of the cancer types. This result is consistent with the assumption that more energy is required for rapid reproduction in cancerous cells. The results also show that two mitochondrial genes, namely MT-RNR2 and MT-CYB are significantly mutated in pan-cancer.

### Stem cell-specific genes

Researches have pointed out a number of similarities between stem cells and cancer cells, including their self-renewal potential and their ability to migrate to other regions of the body [46–48]. Moreover, the ability of stem cells to differentiate into various types of cells increases the risk of malignant transformations. Accordingly, stem cell-specific gene analysis is expected to provide a foundation for better understanding of their role in cancer.

The stem cell-specific gene set studied in this research (shown in Additional file 1: Table A4), which is first identified by Palmer et al. [49], contains 182 protein-coding genes. To extract candidate stem cell-specific genes associated with each of the 29 cancer types as well as with pan-cancer, statistical analysis was performed and subsequently, in the Bonferroni correction, the parameter *m* was set to 182 × 30. With a corrected *p*-value threshold of $\frac{0.05}{182\times 30}$, 57 stem cell-specific genes were selected as candidates for at least one cancer type. The most significant genes among them are CHEK2 and KMT2C, which are associated with 20 and 18 different cancer types respectively. The other genes are related to at most seven types. Given that some researches have already demonstrated the role of CHEK2 [50, 51] and KMT2C [52] in different cancer types, their identified association with a large number of cancer types is not surprising. *Rectum Adenocarcinoma* and *Lung Squamous Cell Carcinoma* cancers are the only cancer types for which no candidate stem cell-specific gene has been identified. In Table 3, the list of candidate stem cell-specific genes for each cancer type is shown. Similar to Table 2, the numbers in this table also show the percentage of patients in which the genes are mutated.

**Table 3:** Candidate stem cell-specific genes for each cancer type

Cancer Type(Percentage)	Genes(Percentage)
ACC (56.5)	HDAC2 (5.4), ERCC2 (20.7), GARS (38.0), PRR34 (8.7)
BLCA (30.3)	CHEK2 (6.1), ERCC2 (9.7),KMT2C (20.9)
BRCA (8.2)	KMT2C (6.9), PILRB (0.8), HLA_DRB5 (0.7)
CHOL (33.3)	CHEK2 (8.3),KMT2C (25),GIMAP8 (2.8)
COAD (1.5)	HLA_DPA1 (1.5),
ESCA (9.3)	NREP (2.2),BRINP1 (7.7)
GBM (4.4)	CHEK2 (1.8),TSHZ2 (2.5)
HNSC (20.4)	CHEK2 (3.8), LIN28B (1.5), BRINP1 (3.1), KMT2C (12.0), NPR3 (2.7)
KICH.21.2)	DIMT1 (1.5),KMT2C (13.6),HLA_DRB5 (7.6), HLA_DQA1 (3)
LIHC (5)	HTR7 (5)
KIRC (11.1)	DNMT3B (3.1), CHEK2 (2.2), RRAS2 (1.8), NREP (0.7), TNFSF10 (1.3), FYB (2.9), SMARCC2 (3.1), RCSD1 (2), HLA_DRB5 (1.8)
KIRP (7.1)	CHEK2 (5.9), DPH3(1.2)
LGG (9.3)	CHEK2(3.9),HDAC2(1.7),ZBTB20(4.6)
LUAD (42.0)	SPDL1 (1.8), CHEK2 (7.2), TRPC4 (7.2), CDH6 (7.2), GIMAP1 (2.2), KMT2C (17.8), PILRB (2.2), TSHZ2 (6.8), NPR3 (4.6), FYB (5.5)
OV (2.6)	BOD1 (0.9), HAS2 (1.7)
PAAD (57.3)	CHEK2 (17.0), BBS9 (9.4), GARS (5.8), SLC24A1 (9.4), KMT2C (17), SMARCC2 (13.5), NPR3 (8.8), AFTPH (13.5)
PCPG (14.3)	CHEK2 (5.1), NUSAP1 (4.0), KMT2C (5.1), HLA_DRB5(1.1)
PRAD (8.9)	CHEK2(3.5),KMT2C(5.4)
SARC (4.3)	ZNF788 (2.8), BRINP1 (2.0)
SKCM (37.3)	GDF3 (8.0), CCDC90B (4.0), CDH6 (10.7), KMT2C (16.0), GIMAP5 (6.7) ,GIMAP7 (6.7),GIMAP1 (6.7),GIMAP8 (12)
STAD (32.3)	CHEK2 (5.4), SOHLH2 (4.4), BRINP1 (5.9), KMT2C (16.5), TSHZ2(7.2),ZBTB20(9)
TGCT (2.8)	C10orf128 (2.1), HLA_DRB5 (2.1), HLA_DQA1 (1.4)
THCA (4.8)	CHEK2 (1.4), GDF3 (0.8), RIOK2 (0.8), HLA_DRB5 (1.7)
THYM (5.7)	CHEK2 (5.7)
UCEC (9.7)	ATP11C (9.7)
UCS(15.8)	CHEK2(7),KMT2C(10.)
UVM(13.8)	CHEK2(7.5),NUSAP1(5),HLA_DRB5(5)
Pan Cancer(24.3)	CHEK2 (4.1), SOHLH2 (1), BRINP1 (2.2), TRPC4 (2.4), CDH6 (2.1), KMT2C (10.6), PILRB (0.8) ,HLA_DRB5 (1.1), TSHZ2 (2.6), NPR3 (1.7),GIMAP1 (0.9), GIMAP8(1.7),ZBTB20(2.1)

### DNA repair genes

DNA repair genes are responsible for recognizing and correcting damages in the replication of DNA. Hence, mutations in DNA repair genes can be expected to alter the efficiency of repairing mechanism, which in turn can be associated with severe health issues such as cancer. Moreover, it has been reported that DNA repair genes are frequently mutated in cancer [53]. Accordingly, studying mutations within DNA repair genes may be helpful for revealing their role in cancer.

To identify DNA repair genes which are associated with a certain type of cancer, a statistical analysis similar to that performed in previous subsections was applied. 174 known DNA repair genes, reported in [54–56], are shown in Additional file 1: Table A5. By setting the parameter *m* to 174 × 30 in the Bonferroni correction, 27 DNA repair genes were identified as candidate for at least one cancer type. The results show that the most significant DNA repair gene is TP53, which was identified as candidate for 25 cancer types as well as for pan-cancer. This conforms with the previous findings about the crucial role of TP53 mutations in cancer development [57, 58]. This further endorses the reliability of the other results in this study. For each cancer type other than *Testicular Germ Cell Tumors*, at least one candidate DNA repair gene was identified. In particular, *Pancreatic Adenocarcinoma* has eight candidate DNA repair genes, and ATM, TCG, TP53 and CHEK2 are the candidate DNA repair genes for pan-cancer. Table 4 shows the candidate DNA repair genes for each cancer type and the number next to each gene shows the percentage of patients in which this gene is mutated.

**Table 4:** Candidate DNA repair genes for each cancer type

Cancer Type(Percentage)	Genes(Percentage)
ACC (37)	MSH3(6.5),TP53(19.6),ERCC2(20.7)
BLCA (63.6)	ATM (13.6), TP53 (49.8) ,ERCC2 (9.7), CHEK2 (6.1)
BRCA (33.4)	TP53 (33.4)
CHOL (22.2)	TP53 (13.9), CHEK2 (8.3)
COAD (52)	TP53 (52.0)
ESCA (87.9)	TP53 (87.9)
GBM (28.7)	TP53 (28.7)
HNSC (71.6)	TP53 (71.2), CHEK2 (3.8)
KICH (33.3)	TP53 (33.3)
KIRC(10.9)	FANCE (4.4), DDB1 (4.9), RPA1 (2.2), TP53 (4.2), CHEK2 (2.2)
KIRP (17.2)	OGG1 (2.4), MSH3 (4.1), TDG (3.6), TP53 (4.1), CHEK2 (5.9)
LGG (50.4)	TP53 (48), CHEK2 (3.9)
LIHC (32.2)	TP53(32.2)
LUAD (57.8)	ERCC5 (3.3), TP53 (54.7), CHEK2 (7.2)
LUSC (79.2)	TP53 (79.2)
OV (84.8)	TP53 (84.8)
UCEC (34.7)	MSH4 (7.3), TP53 (29)
PAAD (84.2)	ERCC3 (8.8), XPC (9.9), WRN (14.6), TDG (9.9), FAN1 (9.9), EME2(11.1), TP53 (67.3), CHEK2 (17)
PCPG (17.7)	FANCD2 (5.1), ERCC8 (1.1), TDG (7.4), CHEK2 (5.1)
PRAD (19.3)	ATM (4.5), TP53 (10.8), POLI (1.4), CHEK2 (3.5)
READ (67.2)	TP53 (67.2)
SARC(37.0)	TP53 (37)
SKCM(22.7)	BLM (6.7), MPG (4.0), TP53 (10.7), CHEK2 (09.3)
STAD (57.9)	UVSSA (4.4), SLX4 (6.7), TP53 (49.9), CHEK2 (5.4)
THCA (4.5)	SMUG1 (0.8), TDG (2.2), TP53 (0.8), CHEK2 (1.4)
THYM (5.7)	CHEK2 (5.7)
UCS (91.2)	TP53 (91.2)
UVM(20)	FANCD2 (6.3), CCNH (2.5), TDG (3.7), CHEK2(7.5)
Pan Cancer(45.2)	ATM(5.5),TDG(1.7),TP53 (39.1),CHEK2 (4.0)

**Fig. 1:**
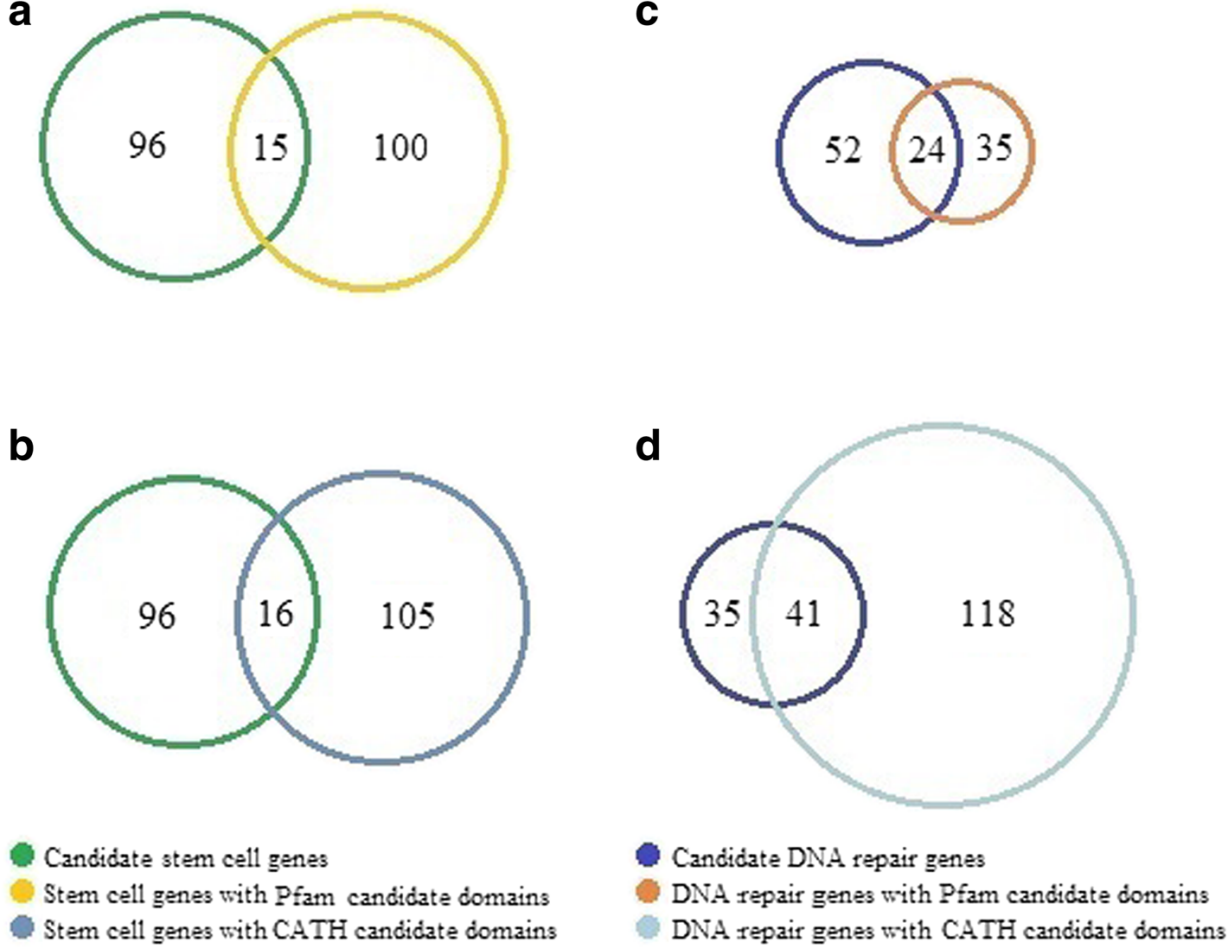
Comparison of candidate genes and genes with candidate domains. (**a**) Comparison of stem cell genes and genes with Pfam candidate domains. (**b**) Comparison of stem cell genes and genes with CATH candidate domains. (**c**) Comparison of DNA repair genes and genes with Pfam candidate domains. (**d**) Comparison of DNA repair genes and genes with CATH candidate domains

To identify cancer-associated genes within mitochondrial, stem cell-specific and DNA repair genes, not only the mutations on domain regions but all those on full protein coding regions are included in the assessment. To be more confident in extracting cancer-associated genes within each biological process, its related candidate genes were restricted to those which also contain at least one candidate domain. Upon studying the mitochondrial genes, we found no candidate domains (defined in the following sections) associated with those genes. Among candidate stem cell-specific genes, 51% and 46% of them contain at least one Pfam and one CATH candidate domain, respectively, as shown in Fig. 1a and b. For each cancer type, the entire list of stem cell-specific genes with Pfam and CATH candidate domains are presented in Additional file 1: Tables A6 and A7. Similarly, 25% and 26% of candidate repair genes consist of at least one Pfam and one CATH candidate domain, respectively, as shown in Fig. 1c and d. More details on repair genes with Pfam and CATH domains are given in Additional file 1: Tables A8 and A9.

### CATH candidate domains

A key objective of this study is to identify CATH candidate domains, which have gone unnoticed in the previous researches conducted in this field. There are 759 CATH-reported domains which are located in 2993 human proteins. Detailed information for each CATH-reported domain can be found in Additional file 1: Table A10. In addition, the position of each CATH domain on each protein-coding gene is available in Additional file 2: Table B1.

To assess CATH domains, the significance level of $\frac{0.05}{30\times 759}$ was used. The results indicate that each cancer type has a number of associated CATH candidate domains ranging from 1 to 19, while pan-cancer analysis reveals 93 related CATH candidate domains. Some domains seemed to not be associated with any individual cancer type, yet they were identified as significant candidates in the pan-cancer study. We say a candidate domain “covers” a particular patient, if the patient has at least one mutation in that specific candidate domain. Surveying the results, we realize that each CATH candidate domain of each cancer type covers various percentages of patients in that cancer type, ranging from 0.02% to 95%. Moreover, all CATH candidate domains of each cancer type cover 28% to 98% of patients of that cancer type. The CATH candidate domains identified for *Breast Invasive Carcinoma*, *Ovarian Serous Cyst Adenocarcinoma* and pan-cancer are presented in Table 5. The number next to each domain shows the percentage of patients which are covered by this domain. Additional file 1: Table A11 shows CATH candidate domains in each cancer type. To assess the statistical significance of an identified candidate domain, the percentage of patients covered by that domain can theoretically be used as a selection attribute.

**Table 5:** Candidate domains for Breast Invasive Carcinoma and Ovarian Serous Cystadenocarcinoma

Cancer Type (Percentage)	CATH Domains (Percentage)
BRCA (77.3)	1.10.1070.11 (33.88), 1.10.220.60 (0.31), 1.10.437.10 (1.73), 1.10.510.10 (25.84), 2.170.260.10 (0.71), 2.40.250.10 (2.03), 2.60.200.10 (1.83), 2.60.40.10 (20.24), 2.60.40.1110 (4.27), 2.60.40.60 (4.48), 2.60.40.720 (33.27)4.10.365.10 (0.71)
OV (80)	1.10.287.650 (0.87), 2.60.40.720 (80.00), 3.30.450.40 (0.87)
Pan Cancer (91.5)	1.10.10.10 (7.90), 1.10.10.440 (0.92), 1.10.10.60 (4.23), 1.10.101.10 (1.59), 1.10.1070.11 (15.52), 1.10.1300.10 (5.87), 1.10.1380.10 (2.34), 1.10.150.210 (0.78), 1.10.150.50 (3.03), 1.10.150.60 (1.30), 1.10.1520.10 (0.55), 1.10.1540.10 (1.47), 1.10.167.10 (3.70), 1.10.246.10 (2.17), 1.10.287.450 (0.94), 1.10.437.10 (2.25), 1.10.472.10 (4.68), 1.10.490.10 (2.46), 1.10.506.10 (0.78), 1.10.510.10 (44.89), 1.10.555.10 (3.85), 1.10.565.10 (10.70), 1.10.630.10 (10.98), 1.10.640.10 (0.98), 1.10.720.50 (0.64), 1.10.750.10 (3.32), 1.10.800.10 (1.60), 1.20.1050.10 (4.98), 1.20.1250.10 (5.37), 1.20.1260.10 (1.29), 1.20.1280.50 (1.17), 1.20.1340.10 (1.61), 1.20.245.10 (0.95), 1.20.5.100 (1.17), 1.20.5.110 (0.48), 1.20.5.50 (1.86), 1.20.58.60 (2.32), 1.20.82.10 (1.01), 1.20.920.10 (6.57), 1.20.930.40 (4.19), 1.25.10.10 (8.64), 1.25.40.20 (8.26), 2.10.220.10 (7.52), 2.10.25.10 (6.69), 2.10.310.10 (0.46), 2.10.60.10 (1.76), 2.10.70.10 (6.31), 2.130.10.10 (7.43), 2.120.10.80 (1.91), 2.140.10.30 (3.99), 2.130.10.130 (3.15), 2.170.270.10 (4.18), 2.170.8.10 (1.20), 2.30.30.190 (1.21), 2.30.39.10 (8.76), 2.30.42.10 (8.87), 2.40.128.20 (4.23), 2.40.20.10 (1.94), 2.40.250.10 (0.38), 2.40.50.40 (4.42), 2.60.120.200 (4.42), 2.60.120.260 (4.65), 2.60.20.10 (2.49), 2.60.200.10 (3.99), 2.60.210.10 (2.78), 2.60.40.10 (33.88), 2.60.40.1110 (6.69), 2.60.40.1120 (1.57), 2.60.40.60 (2.32), 2.60.40.720 (36.55), 2.60.60.20 (1.59), 2.70.98.20 (2.38), 2.80.10.50 (5.22), 3.10.100.10 (5.87), 3.10.20.230 (0.94), 3.10.200.10 (3.60), 3.10.50.10 (2.64), 3.10.620.10 (0.44), 3.20.20.100 (4.55), 3.20.20.140 (4.49), 3.30.1370.10 (2.34), 3.30.1490.20 (2.13), 3.30.300.30 (1.70), 3.30.450.40 (0.48), 3.30.450.50 (0.87), 3.30.70.1230 (0.88), 3.30.70.1470 (0.91), 3.30.70.330 (12.00), 3.30.800.10 (1.98), 3.30.9.10 (0.77), 3.40.190.10 (3.68), 3.40.50.10140 (1.54), 3.40.470.10 (1.13), 3.40.50.10190 (4.22), 3.40.50.1370 (0.75), 3.40.50.2300 (1.51), 3.40.50.300 (25.67), 3.40.718.10 (5.78), 3.90.1170.10 (0.43), 3.90.1230.10 (1.63), 3.90.190.10 (13.32), 4.10.280.10 (1.08), 4.10.365.10 (0.34), 4.10.75.10 (0.72)

### Pfam candidate domains

There are 6009 predicted Pfam domains located in 17,722 human proteins. Detailed information for Pfam domains can be found in Additional file 1: Table A12. In addition, the position of each Pfam domain on each protein-coding gene is available in Additional file 2: Table B2. The significance level of $\frac{0.05}{30\times 6009}$ was used to perform statistical assessment, the results of which show that each cancer type has a different number of Pfam candidate domains, ranging from 3 to 93. For pan-cancer, the number of identified Pfam candidate domains is 202, which indicates a large number of domains are significant to pan-cancer but not to individual cancer types. The results are consistent with those of CATH domains.

Each Pfam Candidate domain of each cancer type covers different percentages of patients with a minimum of 0.2% and a maximum of 98%. Overall, all Pfam candidate domains of each cancer type cover 74% to 100% of patients of that cancer type. Table 6 shows Pfam candidate domains of *Breast Invasive Carcinoma* and *Ovarian Serous Cyst Adenocarcinoma* and the number next to each domain shows the percentage of patients, which are covered by this domain. Additional file 1: Table A13 shows Pfam candidate domains in each cancer type. Similar to CATH candidate domains, the percentage of patients covered by a candidate domain can be used as a proper measure. For instance, P53 and tm_4 cover the first and the second highest percentages (42% and 28%) of *Breast invasive carcinoma* patients, respectively, which shows their significant role in this particular cancer.

**Table 6:** Pfam candidate domains for Breast Invasive Carcinoma and Ovarian Serous Cystadenocarcinoma

Cancer Type (Percentage)	Pfam Domains (Percentage)
BRCA (78.5)	7tm_4 (41.51), ATP-synt_A (0.81), Atrophin-1 (2.85), CBF_beta (2.24), COX1 (2.03), COX3 (1.12), Cadherin (23.91), Cytochrom_B_N_2 (1.12), DUF4647 (1.32), FAM219A (0.51), FRG1 (1.32), GATA (3.97), G_path_suppress (1.12), H-K_ATPase_N (0.31), Histone (7.32), NADH5_C (0.92), NADHdh (1.63), Oxidored_q4 (0.71), Oxidored_q5_N (0.81), P53 (28.48), P53_tetramer (2.03), PI3K_C2 (2.54), PI3K_P85_iSH2 (2.34), PI3K_p85B (1.02), PI3Ka (13.22), PTEN_C2 (2.03), Proton_antipo_M (3.56), Runt (2.44), T-box (4.17), TMEM247 (1.12), Tis11B_N (1.02)
OV (88.3)	7tm_4 (49.57), DUF2462 (0.43), DUF4552 (1.30), MRP-S32 (0.87), NtCtMGAM_N (2.17), ODAM (1.30), P53 (72.61), P53_tetramer (4.78), PTCRA (0.87), Sam68-YY (1.30), UPF0054 (0.87)

**Fig. 2:**
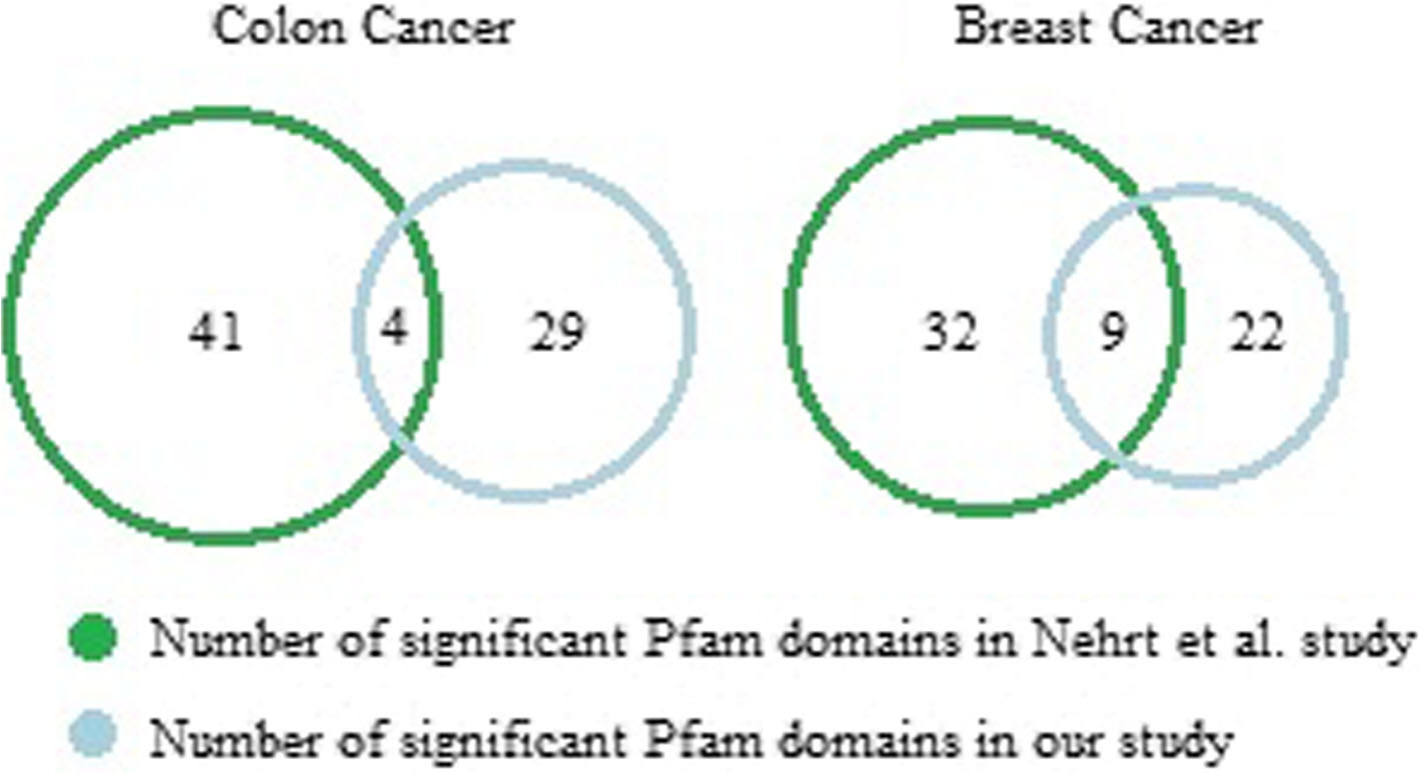
The comparison between our study and Nehrt et al. [13]

The statistical analysis conducted in this study is different to that used by Nehrt et al. [12]. Moreover, different data sources were exploited in these two studies. Therefore, it is no surprise that the results of the two studies are dissimilar. To further emphasize the difference between these approaches, we remark that the number of Pfam domains examined in our study is much larger than that of Nehrt et al. [12] due to the cut-off used in that study for minimum protein or domain length (150 amino acids) and due to Pfam *E*-value threshold used for inclusion (0.001). The comprehensive comparison performed over Pfam and CATH regions (discussed in the next section) clearly indicates the high reliability of Pfam-reported domains, regardless of their associated *E*-values. Furthermore, 5918 out of 6009 investigated Pfam domains have *E*-value less than threshold of 0.001. Also, among 769 identified Pfam candidate domains, 754 (98%) satisfy the threshold condition. Accordingly, we decided not to exclude any Pfam-reported domain. In addition, the statistical method used by Nehrt et al. [12], is extremely sensitive to the number of patients having mutations within the domain region of each protein. This is due to the fact that the number of mutations in each domain is normalized by the cumulative length of all its associated proteins, wherein at least one patient had mutation. Hence, if a new patient with a mutation on an associated protein is added, for which no previous mutation is reported, this would significantly impact the normalizing factor, and subsequently, the statistic used. Moreover, the threshold level of 0.1 is applied in Nehrt et al. [12] for determining significantly mutated domains, by using local false discovery rate (LFDR). As shown in Fig. 2, Nehrt et al. [12] reported 41 and 45 Pfam domains as significantly mutated in *Breast Invasive Carcinoma* and *Colon Adenocarcinoma Tumor*, respectively, while our results identified 31 Pfam candidate domains for *Breast Invasive Carcinoma* and 35 ones for *Colon Adenocarcinoma Tumor*. Comparing the results of the two studies shows that they share nine domains for *Breast Invasive Carcinoma* including CBF_beta, FRG1, GATA, P53, PI3K_p85B, PI3Ka, PTEN_C2, T-box and Tis11B_N. Moreover, the four domains of APC_crr, MH2, P53 and PI3K_p85B are reported by both studies for *Colon Adenocarcinoma Tumor*.

In another study by Yang et al. [10] mutations were obtained from COSMIC database [59] and the analysis was restricted to potentially damaging missense mutations, predicted by IntOGen-mutation platform. To determine significantly mutated domains in a given cancer type, Fisher’s exact test was exploited in that study. Accordingly, the results obtained by Yang et al. [10] are different from those of this study, as expected. The list of cancer types investigated in Yang et al. [11] and those considered in this study share 13 in common. For each of these 13 cancer types, significantly mutated domains obtained by both studies are shown in Table 7. Based on these two studies, seven cancer types share P53 as one of their significant domains.

**Table 7:** Shared significant domains in our study and Yang et al. [11]

Cancer type	Shared domains			
BRCA	Cytochrom_B_C	P53		
COAD	MH2			
ESCA	P53			
HNSC	P53			
KIRC	Bromodomain	Oxidored_q3	VHL	
LIHC	P53			
LUAD	P53	Pkinase_Tyr	Ras	Sushi
LUSC	P53	Sushi		
OV	P53			
PAAD	Ras			
PRAD	MATH			
SKCM	Pkinase_Tyr			
UCEC	DSPc	NADHdh	PI3K_p85B	PI3Ka

### CATH vs. Pfam protein domains

There is a gap between the number of sequenced proteins and that of proteins with known structure, which can also be observed at the level of protein domains. On the other hand, structure-based protein domains are biologically more informative and reliable. Therefore, to benefit from the high number of sequence-based protein domains as well as from the accuracy of structure-based protein domains, both sequence-based and structure-based domains are studied in this research. CATH and Pfam databases are used to extract structure-based and sequence-based domains, respectively.

**Fig. 3:**
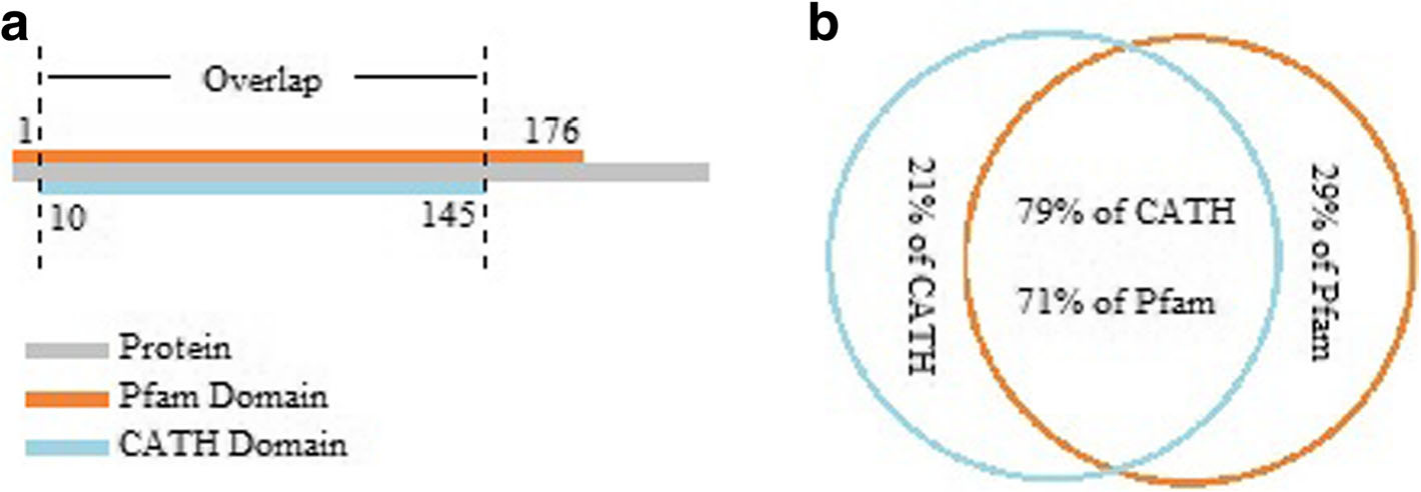
The overlap between Pfam domain region and CATH domain region. (**a**) The overlap between Pfam domain region and CATH domain region for gene VPS25. (**b**) The average overlap between Pfam domain regions and CATH domain regions

Through further investigation, for each protein which has both Pfam and CATH annotations (2974 proteins), the overlap between its Pfam domain region and CATH domain region is computed. For instance, as it is shown in Fig. 3a, for gene VPS25 which contains two homologous domain superfamilies with CATH IDs 1.10.10.10 (amino acids 102–176) and 1.10.10.570 (amino acids 1–176) as well as one Pfam domain of ESCRT-II (amino acids 10–145), the computed overlap is from amino acid 10 to 145. This overlap covers 77% of CATH domain region and 100% of Pfam domain region. Overall, for all 2974 proteins with both Pfam and CATH annotations, computed overlaps cover 79% of CATH domain regions and 75% of Pfam domain regions, on average, as shown in Fig. 3b. This suggests that for a protein with no annotation in CATH database, it is reasonable to study its Pfam domain region as a representative of its functional unit.

**Fig. 4:**
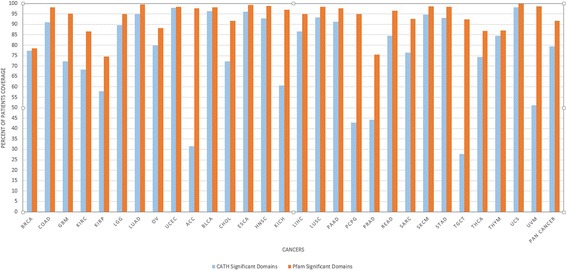
CATH vs. Pfam candidate domain coverage for patients

In addition, the percentage of patients in each cancer type, which are covered by Pfam candidate domains are compared with the ones covered by CATH candidate domains (shown in Fig. 4). As it is shown in Fig. 4, for several cancer types including *Bladder Urothelial Carcinoma*, *Breast Invasive Carcinoma*, *Uterine Corpus Endometrial Carcinoma* and *Uterine Carcinosarcoma*, Pfam and CATH candidate domains cover the same percentage of patients, while in some other types such as *Adrenocortical Carcinoma*, there is a huge gap between the two. The considerably high level of overlap between Pfam and CATH domain regions suggest that wherever CATH candidate domains are incapable of covering patients, Pfam candidate domains are suitable substitutions.

Among 6009 investigated Pfam domains, 769 are identified as candidate domains in at least one cancer type. Candidate domains are observed to be significantly mutated in varying numbers of cancer types (more details are given in Additional file 1: Table A14). To assess the contribution of each candidate domain in different types of cancer, the list of 769 candidate domains were sorted in decreasing order based on the number of associated cancer types. The 17 top-listed domains, presented in Additional file 1: Table A15, are found to be the least number of candidates that each studied cancer type is associated with at least three candidate domains within them. Given that P53 is one of the most commonly mutated domains in all cancers, it is no surprise that it is placed at the top of the list, above other domains. The second domain in the sorted list, tm_4, is identified as a candidate domain for 22 cancer types and for pan-cancer. The tm_4 domain, which is present in a large number of proteins (376), has not previously been implicated in cancer susceptibility, hence can be seen as a newly found candidate.

Proteins of keratin family contain six domains, all except Keratin_assoc are found to be candidate in different numbers of cancer types, ranging from 6 to 17. Interestingly, three of keratin-related domains (Keratin_B2, Keratin_B2_2 and Keratin_2_tail) are placed in our list of top 17 domains. The great contribution of keratin-related domains to cancer may be due to their role in protecting epithelial cells from damage or stress [60].

Similar investigations performed on CATH domains show that among 759 CATH domains, 181 are identified as candidate ones. Detailed information on their associated cancer types are given in Additional file 1: Table A16. Going through the sorted list of CATH candidate domains shows that the 15 top-listed domains, presented in Additional file 1: Table A17, are found to be the least number of candidates that each studied cancer type is associated with at least one candidate domain within them.

Besides, this study sheds some light on the role of domains in cancer. For instance, there are in total 181 CATH and 769 Pfam candidate domains associated to at least one cancer type or to pan-cancer. 94% of Pfam domains and 95% of CATH domains have mutations in more than 95% of their corresponding proteins. However, a high percentage of proteins with mutations on a particular domain does not necessarily imply that domain as a significant candidate. As an example, Pkinase is a domain involved in 348 proteins, for which the number of occurrences on those proteins is 369. Based on the data available, the total number of mutations on this domain in different cancer types is 346, yet it is not identified as a candidate domain for any cancer type. In contrast to Pkinase, Phostensin_N is a domain which is located on two different proteins with mutations on only one of them, yet it is identified as a candidate domain for *Pancreatic Adenocarcinoma*.

Further investigation was performed to determine specific domains of each cancer type. As an example, there are 30 Pfam candidate domains for *Breast Invasive Carcinoma* among which four are only specific to this cancer type, including Atrophin-1, CBF_beta, GATA and Runt. The entire list of specific domains for all cancer types is available in Additional file 1: Table A18. Subsequently, a protein is termed “Pfam specific protein” to a cancer type, if it contains only those Pfam candidate domains which are specific to the cancer of interest. Additional file 1: Table A19 shows all proteins with mutations on Pfam candidate domains and Additional file 1: Table A20 presents Pfam specific proteins of all cancer types. Similar tables (Additional file 1: Tables A21, A22 and A23) are provided for CATH candidate domains. For each cancer type, the number of Pfam candidate domains, proteins with mutations on Pfam candidate domain, Pfam specific domains and specific proteins are summarized in Table 8. Table 9 shows similar results for CATH candidate domains. On average, the number of CATH specific domains for a cancer type is 2.4, whereas this number is 15.8 for Pfam specific domains.

**Table 8:** Number of Pfam Candidate domains, proteins with candidate domains and specific domains and proteins for each cancer type

Cancer type	Number of Pfam candidate domains	Number of Proteins with Pfam candidate domains	Number of Pfam specific candidate domains	Number of Pfam specific proteins
ACC	57	323	23	47
BLCA	51	1076	15	64
BRCA	31	495	4	11
CHOL	39	45	19	21
COAD	35	955	6	7
ESCA	25	355	4	5
GBM	24	474	6	8
HNSC	42	743	5	42
KICH	55	200	21	23
KIRC	90	494	54	157
KIRP	36	84	22	44
LGG	25	366	3	15
LIHC	25	432	5	9
LUAD	115	2328	27	248
LUSC	31	1128	5	8
OV	11	188	6	6
PAAD	143	793	70	151
PCPG	70	151	20	44
PRAD	34	353	13	20
READ	29	266	10	17
SARC	25	383	7	11
SKCM	40	1116	11	25
STAD	52	1476	10	35
TGCT	66	366	29	43
THCA	38	304	15	19
THYM	18	41	5	5
UCEC	23	1094	3	16
UCS	44	57	19	27
UVM	63	99	22	34
Pan-cancer	222	3685	27	315

**Table 9:** Number of CATH Candidate domains, proteins with candidate domains and specific domains and proteins for each cancer type

Cancer type	Number of CATH candidate domains	Number of proteins with CATH candidate domains	Number of CATH specific candidate domains	Number of CATH specific proteins
ACC	7	15	4	5
BLCA	22	649	1	1
BRCA	12	315	1	2
CHOL	9	39	2	4
COAD	16	377	0	0
ESCA	13	191	2	2
GBM	9	28	0	0
HNSC	14	372	0	0
KICH	10	18	6	7
KIRC	19	49	3	3
KIRP	11	110	8	10
LGG	7	21	1	2
LIHC	14	307	2	4
LUAD	32	590	3	24
LUSC	11	154	0	0
OV	3	6	1	2
PAAD	20	249	6	27
PCPG	9	56	3	3
PRAD	13	113	4	5
READ	7	116	0	0
SARC	8	285	1	2
SKCM	17	281	5	16
STAD	26	575	2	6
TGCT	9	78	3	5
THCA	5	91	1	3
THYM	5	146	2	11
UCEC	26	449	4	5
UCS	11	22	3	4
UVM	6	13	2	2
Pan-cancer	104	456	18	48

**Fig. 5:**
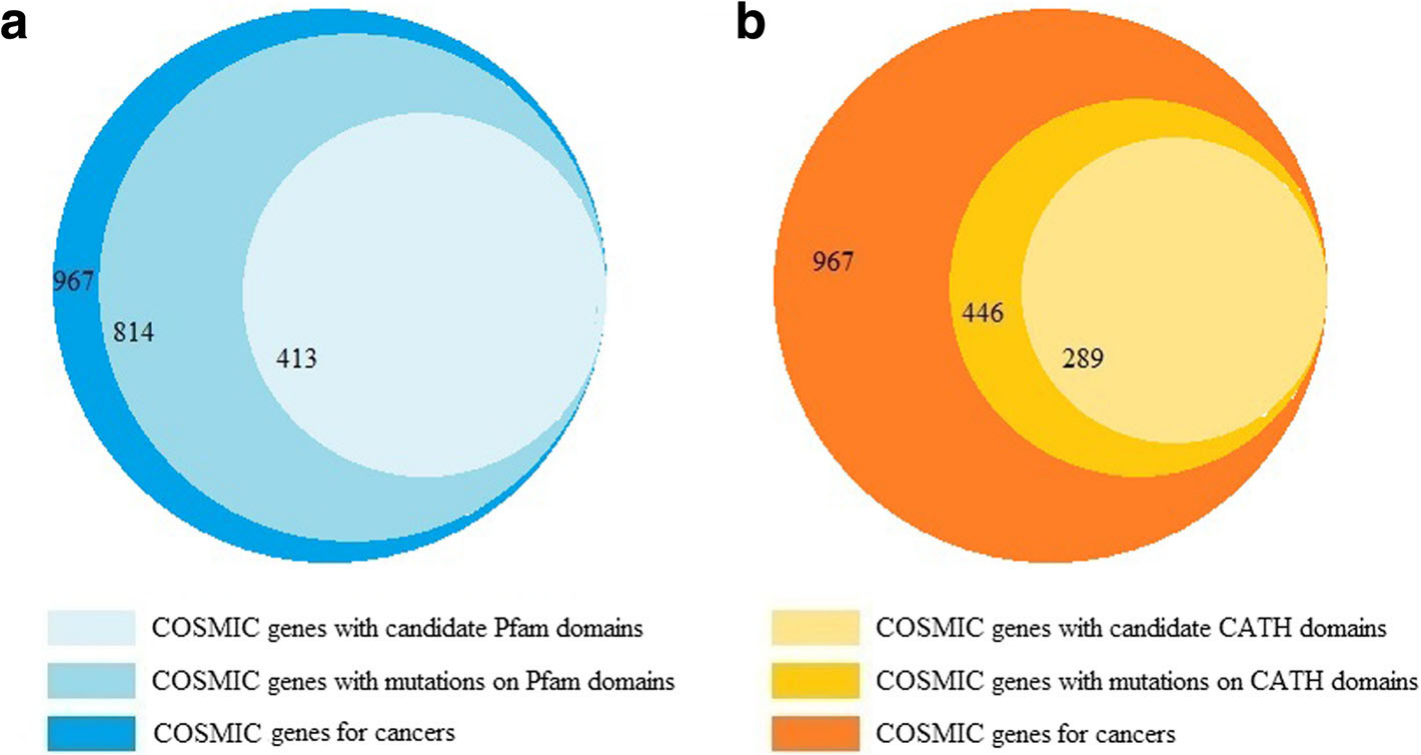
Comparison of causal genes in COSMIC with genes having candidate domains. (**a**) Comparison of COSMIC genes with genes having Pfam candidate domains. (**b**) Comparison of COSMIC genes with genes having CATH candidate domains

To evaluate the reliability of the proposed methodology, the following process was performed. For each cancer type, the genes that contain identified candidate domains and have mutations in that cancer were compared with experimentally verified cancer genes from COSMIC [59]. A list of 616 unique genes for which mutations have been causally implicated in cancer, were downloaded from COSMIC database (in May 2017). Since some of the cancer causal genes are associated to more than one cancer type, the cumulative number (counting with repetition) of genes, for all 29 cancer types investigated in this study tallied 967. Considering all 29 cancer types (shown in Fig. 5), 814 COSMIC genes have mutations on Pfam domains, out of which, 413 genes contain at least one Pfam candidate domain. Therefore, among COSMIC genes with at least one Pfam domain, 51% have Pfam candidate domains. For each cancer type, the list of its specific COSMIC genes which also have Pfam candidate domain is given in Additional file 1: Table A24.

Similar analysis for CATH domains indicates that among 967 cancer causal genes, there are 446 genes with mutations on their CATH domains, out of which, 289 genes have mutations on CATH candidate domains. Detailed information for each cancer type is presented in Additional file 1: Table A25. Even though 52% of the cancer causal genes reported by COSMIC have no CATH domains, among those with at least one CATH domain, 65% have the candidate ones. This suggests that CATH domains are superior indicators compared to Pfam domains, in identification of cancer causal genes.

Curiously, studying mutations at domain level provides an interesting insight into the role of gene families in cancer development. For instance, in *Uterine Corpus Endometrial Carcinoma*, eight out of nine genes in TCEAL gene family have mutations on identified Pfam candidate domains. Members of this family had already been identified as nuclear phosphoproteins that modulate transcription in a promoter context-dependent manner [61]. Besides, for 19 out of 22 genes of FGF gene family, mutations occur on Pfam candidate domains in *Stomach adenocarcinoma*. FGF’s are known to play a key role in the processes of proliferation and differentiation of a wide variety of cells and tissues [62].

We also evaluated the impact of domain mutations in cancer using SnpEff [63], which annotates the effects of variants on genes and classifies them as low, moderate and high impact. The results indicate that 13.4% of mutations on Pfam and CATH domains are classified as high impact mutations and the rest are reported as moderate ones. Restricting the analysis to candidate domains shows that 14.3% and 17.7% of mutations on Pfam and CATH candidate domains are considered as high impact ones, respectively. Moreover, we calculated the frequency of different types of variants, including nucleotide variants, insertions and deletions, on domain regions. Our analysis reveals that all variants has similar frequencies on domains and candidate domains.

### Protein-protein interactions

Protein-Protein Interactions (PPIs) commonly refer to physical contact between two or more proteins [64] and offer a wealth of molecular information, which exists in various molecular pathways. Besides, domains are usually responsible for mediating protein-protein interactions [65]. Understanding specific interaction map of a disease is becoming increasingly crucial in elucidating its underlying molecular mechanisms. Therefore, it is reasonable to study PPI for proteins with candidate domains. To this end, the specific proteins of each cancer type are exploited to determine specific PPIs of that cancer type. The interactome database STRING [66] was used to extract all interactions with the highest confidence score (0.9 and above) among specific proteins of each cancer type. The results show that in 20 out of 29 cancer types and also in pan-cancer, specific proteins are significantly connected. The connectivity significance of proteins was determined by the PPI enrichment *p*-value, reported by STRING. This measure quantifies whether the set of input proteins have more interactions among themselves than a random set of proteins of similar size.

Subsequently, for each cancer type in which a significant interaction network is not formed on its specific proteins, the same analysis was performed on the entire list of proteins with mutation on Pfam candidate domains of that cancer type. The entire lists of proteins are significantly interconnected for all cancer types other than *Ovarian serous cystadenocarcinoma*. Table 10 gives some information about the number of nodes and edges in the interaction networks of each cancer type, as well as their enrichment *p*-values. Furthermore, for each cancer type the interactome analysis was performed on the entire list of proteins with mutation on CATH candidate domains of that cancer type. The results show that in all cancer types except *Uveal Melanoma*, proteins with mutations on CATH candidate domains are significantly connected. Some information including the number of nodes and edges in each interaction network, and its enrichment *p*-value is presented in Table 11. Due to the small number of proteins in the CATH specific lists, interactome analysis was not performed on them.

**Table 10:** The result of interactome analysis for Pfam candidate domains in different cancer types

Cancer type	Pfam specific proteins					Proteins with Pfam candidate domain				
	#Nodes	#Edges	Expected number of edges	PPI enrichment *p*-value	Significant or Not	#Nodes	#Edges	Expected number of edges	PPI enrichment *p*-value	Significant or Not
BRCA	16	14	5	0.002	YES					
COAD	7	1	0	0.04	YES					
GBM	8	1	0	0.126	NO	471	2687	558	0	YES
KIRC	156	40	16	4E-07	YES					
KIRP	43	30	3	0	YES					
LGG	14	2	0	7E-04	YES					
LUAD	247	282	9	0	YES					
OV	6	0	0	1	NO	186	3	1	0.0738	NO
UCEC	16	1	0	7E-04	YES					
ACC	46	3	1	0.069	NO	319	113	14	0	YES
BLCA	64	14	2	1E-07	YES					
CHOL	21	4	0	2E-04	YES					
ESCA	5	1	0	0.002	YES					
HNSC	42	25	1	0	YES					
KICH	23	1	2	0.79	NO	198	105	33	0	YES
LIHC	9	1	0	0.016	YES					
LUSC	8	0	0	1	NO	1132	9242	2583	0	YES
PAAD	151	59	21	6E-12	YES					
PCPG	43	5	1	0.01	YES					
PRAD	20	7	1	2E-06	YES					
READ	17	4	0	1E-04	YES					
SARC	11	3	0	2E-05	YES					
SKCM	25	22	4	7E-11	YES					
STAD	35	69	1	0	YES					
TGCT	42	8	1	3E-06	YES					
THCA	19	1	0	0.149	NO	301	132	29	0	YES
THYM	5	0	0	1	NO	40	32	0	0	YES
UCS	26	1	1	0.462	NO	55	45	8	0	YES
UVM	34	3	3	0.609	NO	98	22	14	0.039	YES
Pan-cancer	312	231	34	0	YES

**Table 11:** The result of interactome analysis for CATH candidate domains in different cancer types

Proteins with at least one CATH candidate domain					
Cancer type	#Nods	#Edges	Expected number of edges	PPI enrichment *p*-value	Significant or not
BRCA	315	1136	261	0	*YES*
COAD	377	1325	363	0	*YES*
GBM	28	61	9	0	*YES*
KIRC	49	37	13	1.62E-08	*YES*
KIRP	110	199	57	0	*YES*
LGG	21	26	4	2.55E-13	*YES*
LUAD	588	2173	652	0	*YES*
OV	6	3	0	0.000256	*YES*
UCEC	449	2159	671	0	*YES*
ACC	15	1	0	0.152	*YES*
BLCA	648	3067	1487	0	*YES*
CHOL	39	21	6	4.84E-06	*YES*
ESCA	191	703	180	0	*YES*
HNSC	370	1322	366	0	*YES*
KICH	18	15	2	7.81E-10	*YES*
LIHC	307	1065	292	0	*YES*
LUSC	154	177	40	0	*YES*
PAAD	249	610	231	0	*YES*
PCPG	56	35	10	5.99E-10	*YES*
PRAD	113	249	72	0	*YES*
READ	116	285	68	0	*YES*
SARC	285	902	223	0	*YES*
SKCM	281	498	145	0	*YES*
STAD	575	2181	762	0	*YES*
TGCT	78	91	9	0	*YES*
THCA	91	248	136	0	*YES*
THYM	146	411	83	0	*YES*
UCS	22	15	6	0.00156	*YES*
UVM	13	3	1	0.186	***NO***
Pan-cancer	457	1821	610	0	*YES*

Overall, there are 4968 proteins with mutations on Pfam candidate domains, each of which is associated with 4 cancer types on average. About 31% of them are specific to only one cancer type and more than 7% of them (353 proteins) are linked to at least half of cancer types. Given these figures, one can expect the specific proteins to be not significantly interconnected in some cancer types, even though their entire list of proteins with mutations on candidate domains form highly connected networks. Additional file 1: Table A26 presents the number of associated cancer types for each protein. To have a more comprehensive picture of CATH candidate domains, similar results for 1379 proteins with mutations on CATH candidate domains are given in Additional file 1: Table A27.

### Website

**Fig. 6:**
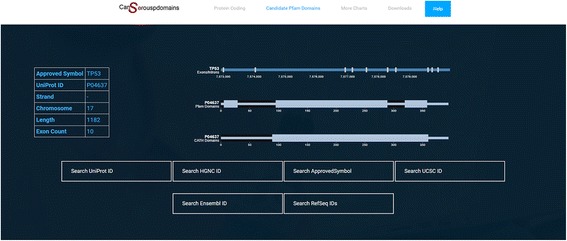
The result of searching TP53 in website

As previously noted, data integration is an essential requirement for this study and related fields of research. Several data sources were used to incorporate different types of information for human protein-coding genes, including gene symbols and protein identifiers from multiple resources, the start and end positions of each CATH and Pfam domain within a given protein, and the positions of exons and introns in the human genome. This comprehensive data integration provides researchers with a unified data source, which can be accessed via http://www.cancerouspdomains.ir, as well as from Additional file 2: Table B3. All previously mentioned tables in Additional file 1 are also downloadable from the website. An example of using the embedded search engine to extract the integrated information for gene TP53 is depicted in Fig. 6.

**Fig. 7:**
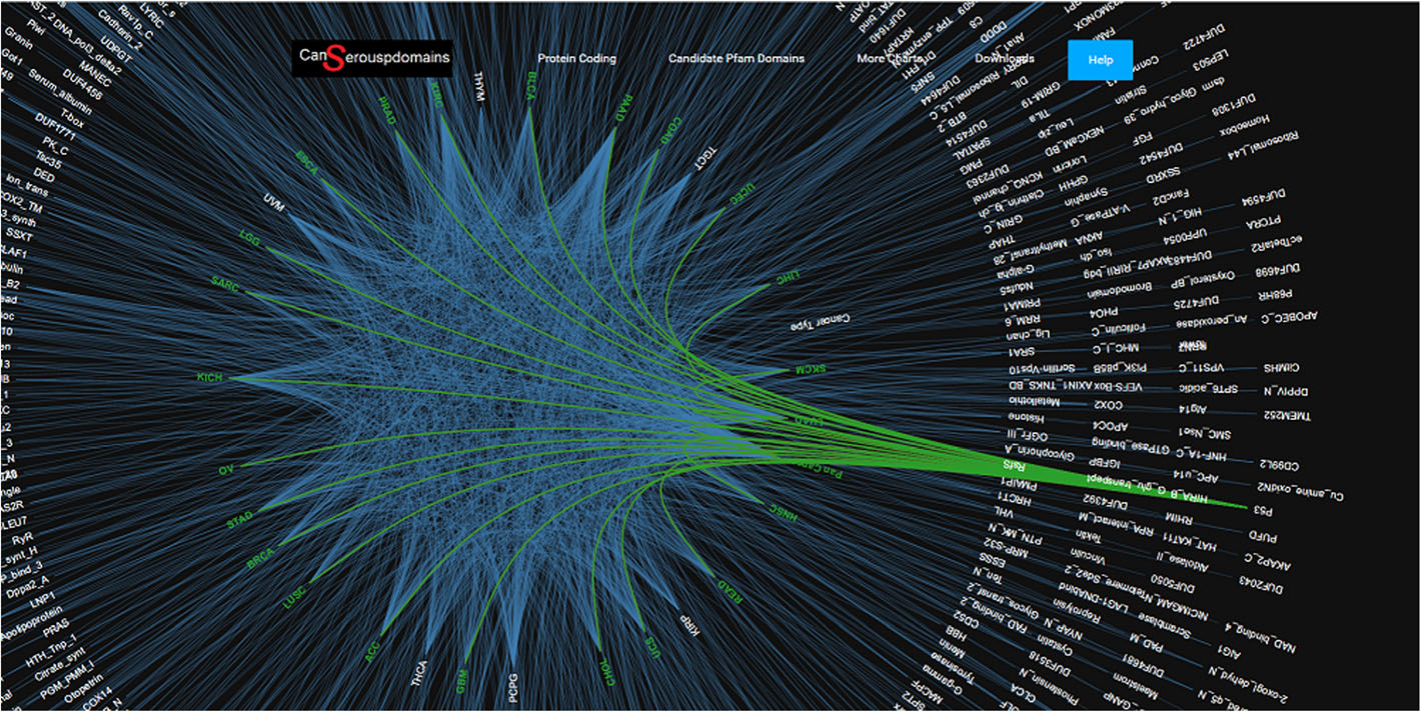
The graph chart for Pfam candidate domains

Some of the highly beneficial data provided on the website are the five graph charts, which show the associations between different groups of genes or domains to various cancer types. These graph charts are represented by bipartite graphs in which one set of nodes corresponds to the genes/domains and the other set of nodes corresponds to cancer type. In each bipartite graph, an edge connects a gene/domain to a cancer type, if this gene/domain is identified as a candidate in the cancer type. For instance, one set of nodes represents Pfam candidate domains and the other set represents different cancer types. An edge connects two nodes in these sets, if the corresponding domain is a candidate for the corresponding cancer type. The graph chart of Pfam candidate domains is illustrated in Fig. 7.

A help file is also provided for detailed description of the information embedded in the website. Some tailored interface options such as “moving”, “zoom in” and “zoom out” are available to control the size of the display. These options are specifically helpful due to the large number of CATH and Pfam candidate domains. Furthermore, by clicking on each cancer type, all related candidate domain or genes can be distinguished via a change of edge colors. The same option is also provided for each candidate domain or gene.

## Conclusion

Distinguishing mutations on protein-coding regions which impact the functionality of coded proteins, is one of the main obstacles in the study of cancer genomics. The exome-wide study of somatic mutations for several cancer types and the available structural information of proteins provide invaluable resources for specifically studying cancer genomics at functional level. Both sequence-based and structure-based domains, which are available in Pfam and CATH databases respectively, are promising representatives of functional regions within proteins. Accordingly, extracting domain regions which are significantly mutated in a cancer type reveals critical information required for validating the impact of mutations.

In this paper, a comprehensive investigation is conducted on all 29 TCGA cancer types and pan-cancer to identify sequence-based and structure-based domains in which mutations occur with high statistical significance. The domains identified for each cancer type offer an explanation for the functional impact of mutations in that cancer type. It is shown that each cancer type has its specific set of candidate domains, which in turn suggests a specific set of proteins associated to that cancer type. The interactome analysis showed that the specific proteins of each cancer type are significantly connected. This interconnectivity supports the idea that leveraging domain regions can improve accurate identification of functionally related causal proteins. Through further investigation, the frequency of mutations in mitochondrial genes, stem cell-specific genes and DNA repair genes was determined to examine their role in cancer development and progression.

Additionally, this study provides other researchers with a comprehensive and unified data repository for studying both CATH and Pfam domain regions on protein-coding genes. Moreover, it has clarified the associations between different groups of genes or domains and various cancer types. All this information is accessible via our website.

## Additional files

Additional file 1: It contains 27 supplementary tables, Table A1–27 with the data described in the text. (XLSX 1032 kb)

Additional file 2: It contains three tables, Tables B1-B3 with the data gathered from different datasets. All these data are used in study. (XLSX 55782 kb)

## Abbreviations

HUGO: HUman Genome Organization
TCGA: The Cancer Genome Atlas
UCSC: University of California Santa Cruz
